# IGFLR1 as a Novel Prognostic Biomarker in Clear Cell Renal Cell Cancer Correlating With Immune Infiltrates

**DOI:** 10.3389/fmolb.2020.565173

**Published:** 2020-11-26

**Authors:** Wenjing Song, Youcheng Shao, Xin He, Pengju Gong, Yan Yang, Sirui Huang, Yifan Zeng, Lei Wei, Jingwei Zhang

**Affiliations:** ^1^Department of Breast and Thyroid Surgery, Zhongnan Hospital, Hubei Key Laboratory of Tumor Biological Behaviors, Hubei Cancer Clinical Study Center, Wuhan University, Wuhan, China; ^2^Department of Pathology and Pathophysiology, School of Basic Medical Sciences, Wuhan University, Wuhan, China

**Keywords:** IGFLR1, myeloid derived suppressor cell, tumor-infiltrating, prognosis biomarker, kidney clear cell carcinoma

## Abstract

**Objective:**

Insulin Growth Factor-Like receptor 1 (IGFLR1) reflects progressive disease and confers a poor prognosis in clear cell renal cell cancer (ccRCC). However, extensive studies highlighting the mechanisms involved in how IGFLR1 triggers the progression of ccRCC remain lacking.

**Methods:**

In the present study, the expression level of IGFLR1 mRNA and correlation between IGFLR1 expression and prognosis of ccRCC were analyzed based on The Cancer Genome Atlas (TCGA) ccRCC cohort. Further, we analyzed methylation and copy number variation to try to explain the difference in IGFLR1 expression. Subsequently, we investigated the correlation between IGFLR1 and tumor-infiltrating immune cells with the aid of TIMER (Tumor Immune Estimation Resource). The potential candidates’ genes associated with IGFLR1 were screened by variation analysis, which were used for further enrichment analysis of signaling pathways and immune gene sets to infer the certain function and corresponding mechanisms in which IGFLR1 was involved in ccRCC. Finally, we establish prognostic risk models using multivariate Cox regression analysis and analyzed the possible involvement of IGFLR1 in chemotherapeutic drug resistance.

**Results:**

The results showed that upregulated IGFLR1 was detected in ccRCC compared with para-cancer tissues and significantly affected the prognosis of ccRCC (overall survival: Logrank *p* < 0.0001; disease free survival: Logrank *p* = 0.022). Univariate and multivariate analyses indicated that IGFLR1 was an independent prognostic factor for ccRCC (HR = 2.064, *p* = 0.006) and the risk prognostic model based on age, M, level of platelet and calcium and IGFLR1 expression had satisfying predictive ability. The correlation analysis showed that the expression level of IGFLR1 was positively correlated with the abundance of myeloid derived suppressor cell and their marker genes in ccRCC significantly. IGFLR1 may be related to the regulatory activation, intercellular adhesion of lymphocytes and drug resistance in cancer.

**Conclusion:**

These findings suggested that IGFLR1 was significantly associated with the prognosis in a variety of cancers, particularly ccRCC. IGFLR1 may play an important role in tumor related immune infiltration and showed potential diagnostic, therapeutic and prognostic value in ccRCC.

## Introduction

Renal cell carcinoma is one of the most common malignancies worldwide, accounting for 4.2% of all new cancer cases, with an estimated 73,750 new cases and 14,830 deaths in the United States in 2020 (Siegel et al., 2019). Clear cell renal cell carcinoma (ccRCC) is the most common type of renal cancer, accounting for 85% of all renal cancers (Choueiri and Motzer, 2017). In most cases, radiotherapy and chemotherapy have a poor effect on ccRCC, so surgical operation remains the primary treatment for ccRCC. As a heterogeneous disease, ccRCC still lacks effective biomarkers for individualized treatment options, especially in current immunotherapy (Barata and Rini, 2017). Therefore, there is an urgent need to discover novel immune-related molecular biomarkers and therapeutic targets of ccRCC.

The interaction between tumor cells and the immune system plays a significant role in the occurrence, development and treatment of cancer. The tumor microenvironment (TME) consists of tumor cells and their surrounding stroma, innate immune cells and adaptive immune cells. Tumor infiltrating immune cells (TIICs), which forms an ecosystem in the TME, can be regulated by different cytokines and chemokines to control or promote tumor growth, regulate cancer progression and demonstrate potential prognostic value (Grivennikov et al., 2010).

Insulin Growth Factor-Like receptor 1 (IGFLR1), also known as transmembrane protein 149 (TMEM149), is a protein encoding gene located on chromosome 19 and is widely expressed in lymph nodes, spleen and kidney (Fagerberg et al., 2014). Although studies have shown that high expression level of IGFLR1 could predict poor survival among patients with ccRCC (Parker et al., 2002; Sichani et al., 2010; Lkhagvadorj et al., 2014), the mechanism of the cancer-promoting effect of IGFLR1 has been still unclear, and previous studies were limited to carbohydrate metabolism (Solarek et al., 2015). Adrian et al. found that IGFLR1 was similar in structure to the tumor necrosis factor receptor family (TNFRs) and considered to be a novel receptor in the TNFRs (Lobito et al., 2011). TNFRs was most widely expressed in the immune system and had a regulatory effect on both congenital and adaptive immunity. IGFLR1 was expressed on the surface of T cells of mouse, and its high expression enhances inflammatory infiltration and activation (Lobito et al., 2011). Lineage tracking of T cells in colorectal cancer (CRC) by Zhang et al. (2018) revealed that IGFLR1 was highly expressed in both CXCL13+BHLHE40+ TH1-like cells and CD8+ exhausted T cells and possessed co-stimulatory functions. Ren and Zhang (2019) measured scRNA-seq in colorectal cancer, hepatocellular carcinoma and non-small cell lung cancer and found that IGFLR1 may be a tumor immune-related molecule. However, the potential function and prognostic value of IGFLR1 in tumor progression and tumor immunology remained unclear.

In the present study, we analyzed the expression level in cancer and para-cancer tissues, and investigated the differences in promoter methylation and copy number variation (CNV) of IGFLR1 in ccRCC whereafter. Furthermore, to explore the role of IGFLR1 in tumor immunity, we analyzed the correlation between IGFLR1 and infiltration of TIICs and the immune-related signaling pathways through correlation analysis and GSEA. We hope this study will contribute to new prognostic monitoring and treatment strategies for ccRCC patients.

## Manuscript Formatting

### Methods

#### Patient Data Acquisition

The Cancer Genome Atlas (TCGA)^1^ is a joint cancer research project established by the National Cancer Institute and the National Human Genome Research Institute, covering 33 types of cancer. After data cleaning, we downloaded RNA-Seq and corresponding clinical data (survival time, status, age at diagnosis, race, gender, neoadjuvant treatment or non, laterality, histological grade, pathological T, N, M and stage, level of hemoglobin, counts of white blood cell, platelet and concentration of calcium) of ccRCC patients at last. Data download and online analysis will be available by December 31, 2019 (TCGA: v21.0). The clinical and histopathological characteristics of the ccRCC population were presented in Table 1.

**TABLE 1:** Demographics and clinical pathologic characteristics of ccRCC patients.

Characteristics	*N* = 162	
	N	%
**Age at diagnosis**		
≤60	70	43.21
>60	92	56.79
**Race**		
Asian	2	1.23
Black or African American	4	2.47
White	156	96.30
**Gender**		
Male	96	59.26
Female	66	40.74
Neoadjuvant treatment		
Yes	6	3.70
No	156	96.30
**Laterality**		
Right	83	51.23
Left	79	48.77
**Histological grade**		
Grade1	1	0.62
Grade2	68	41.98
Grade3	64	39.51
Grade4	29	17.90
**Pathological T**		
T1	64	39.51
T2	25	15.43
T3	67	41.36
T4	6	3.70
**Pathological N**		
N0	154	95.06
N1	8	4.94
**M**		
M0	134	82.72
M1	28	17.29
**Stage**		
Stage I	62	38.27
Stage II	20	12.35
Stage III	51	31.48
Stage IV	29	17.90
**Hemoglobin**		
Elevated	1	0.62
Normal	59	36.42
Low	102	62.97
**White blood cell**		
Elevated	63	38.89
Normal	95	58.64
Low	4	2.47
**Platelet**		
Elevated	20	12.35
Normal	121	74.69
Low	21	12.96
**Calcium**		
Elevated	5	3.09
Normal	64	39.51
Low	93	57.41
**Survival status**		
Dead	71	43.83
Survivor	91	56.17

#### The Expression Profile of IGFLR1

Oncomine database^2^ (Rhodes et al., 2007) is a large tumor gene chip database that incorporates GEO, TCGA and published literature. The Tumor immune estimation resources (TIMER) platform^3^ (Li et al., 2017) is a web server for the comprehensive analysis of TIICs in TCGA cancers. We analyzed the expression level of IGFLR1 in cancer and para-cancer tissue through “Gene Summary” module in Oncomine and “Differential Expression” module in TIMER. The results generated in Oncomine are displayed with *p*-values, fold changes, and ranks. In TIMER, distributions of IGFLR1 expression levels are displayed by box plots, with statistical significance of differential expression evaluated using the Wilcoxon test. The Broad Institute-Cancer Cell Line Encyclopedia (CCLE)^4^ provided a collection of 1,036 human cancer cell lines from 36 tumor types. Human Protein Atlas (HPA)^5^ (Uhlén et al., 2015; Thul et al., 2017; Uhlen et al., 2017) provides tissue and cell distribution information of 26,000 Human proteins. In this database, the researchers used highly specific antibodies and immunoassay techniques (Western blotting, immunofluorescence, and immunohistochemistry) to examine in detail the expression of proteins in cell lines, tumor tissues and normal samples. The mRNA and protein expression of IGFLR1 in tissues and cells was analyzed by CCLE and HPA database, respectively.

#### Methylation Profile of IGFLR1

UALCAN^6^ (Chandrashekar et al., 2017) is an interactive web resource for analyzing the cancer transcriptome data based on the TCGA database. We analyzed the IGFLR1 promoter methylation level in ccRCC tissues with different stage and grade via “methylation” module. Median of methylation level were compared using independent group *t* tests when the data were normally distributed; otherwise, the Wilcoxon test was used. MEXPRESS^7^ (Koch et al., 2015, 2019) is tool designed for the easy visualization of the relationship between clinical information, methylation and expression of cancer patients from TCGA. We analyzed the association between IGFLR1 expression, copy number, clinical data and DNA methylation using the MEXPRESS tool. The variable on which the samples are sorted is compared to all the other available variables and based on the type of data (numerical/categorical) an appropriate test is performed, and all *p*-values have been corrected for multiple hypothesis testing. Detailed statistical methods can refer to the website^8^.

#### Transcription Factor-mRNA Interaction Analysis

We used TRRUST (version 2)^9^ (Han et al., 2018), a manually curated database of human and mouse transcriptional regulatory networks derived from 11,237 PubMed articles for Transcription factor (TF)-IGFLR1 interaction analysis.

#### Prognosis Analysis

Gene expression profiling interactive analysis (GEPIA)^10^ (Tang et al., 2017) is an interactive web server containing RNA-Seq expression data of cancer and para-cancer samples from the TCGA. We evaluated the relationship between IGFLR1 expression level and overall survival (OS), disease free survival (DFS) of ccRCC patients via “Survival” module in GEPIA, respectively. The Kaplan-Meier (K-M) method was used to create the survival plots and the log-rank test was used to compare the difference in survival curves (cutoff by median expression level of IGFLR1). In addition, the expression levels of IGFLR1 in ccRCC tumor tissues from TCGA with different histological grade and pathological T, N, M, and stage were compared, subsequently visualized as box plots.

#### Expression and Survival Analysis of IGFL1

The expression of IGFL1, the ligand of IGFLR1, in general carcinoma was analyzed by using TIMER database. Further, ccRCC samples were divided into two groups based on the expression level of IGFLR1. The expression level of IGFL1 in the samples with high expression of IGFLR1 and low expression of IGFLR1 was compared and the influence of the expression level of IGFL1 in the samples with high expression of IGFLR1 on the prognosis of ccRCC patients was analyzed by “survival” R package.

#### Functional Inference of IGFLR1

According to the median expression level of IGFLR1 in ccRCC, the tumor samples were divided into two groups and analyzed the differentially expressed genes (DEGs) in the two groups by “limma” R package, and the results were visualized with volcano plot. To explore the IGFLR1 involved in the regulation of the signaling pathway, Gene Ontology (GO) enrichment and Kyoto Encyclopedia of Genes and Genomes (KEGG) pathway analysis of DEGs was implemented using the “clusterProfiler” R package. Gene Set Enrichment Analysis (GSEA), which can be acquired from the Broad Institute GSEA website^11^ (Mootha et al., 2003; Subramanian et al., 2005), is a desktop software used to analyze gene sets. To infer the immune gene set involved in IGFLR1, immunologic signature gene sets (C7, v6.2) in IGFLR1-high-expression and IGFLR1-low-expression groups were enriched by GSEA (version 4.0.1). The top 5 gene sets enriched, respectively in the high-expression group and the low-expression group were plotted using R language (version 3.6.1).

#### Immunocorrelates of IGFLR1

TISIDB^12^ (Ru et al., 2019), an integrated library portal for tumor-immune system interactions, estimates the association between gene expression and immune function (including lymphocytes and various immune-related factors) based on literature retrieval, analysis of high-throughput screening from TCGA database. First, we analyzed the correlation between IGFLR1 and 28 TIICs in ccRCC via “lymphocyte” module. Further, we searched and screened cell markers for myeloid-derived suppressor cell (MDSC) using the CellMarker^13^. Finally, the correlation between the expression of IGFLR1 and the molecular markers of MDSC was evaluated by Spearman’s correlation and statistical significance on TIMER and TISIDB.

#### Establishment and Evaluation of Prognostic Risk Model

Age at diagnosis, race, gender, neoadjuvant treatment or non, laterality, histological grade, pathological T, N, M and stage, level of hemoglobin, counts of white blood cell, platelet and concentration of calcium were included in the present study. Univariate Cox regression analysis was conducted and variables that were statistically significant (*p* < 0.05) were selected as prognostic factors. Further, multivariate Cox regression analysis was performed on the above factors to establish a prognostic risk model, provide the risk score and plot nomogram. The survival status diagram, risk heatmap and survival curve were drawn based on the risk score. The accuracy and specificity of the evaluation model were quantified through The area under the curve (AUC) with 95% confidence interval was determined from the receiver operating characteristic curves (ROC).

#### Analysis of Drug Sensitivity Associated With IGFLR1

GSCALite^14^ (Liu et al., 2018) is web-based platform for Gene Set Cancer Analysis. We investigated the drug resistance related to IGFLR1-related genes via “drug sensitivity analysis” module based on the integration of Genomics of Drug Sensitivity in Cancer (GDSC).

#### Statistical Analysis

Most of the statistical analysis were performed by online bioinformatics databases and tools as mentioned. Mean and median for continuous variables were compared using independent group t tests when the data were normally distributed; otherwise, the Wilcoxon test was used. Chi-square test is used to compare clinical and pathological parameters and other categorical variables. Tests were two-sided and *p*-value < 0.05 were considered statistically significant.

## Results

### The Expression Level of IGFLR1 in Pan-Cancers

**FIGURE 1:**
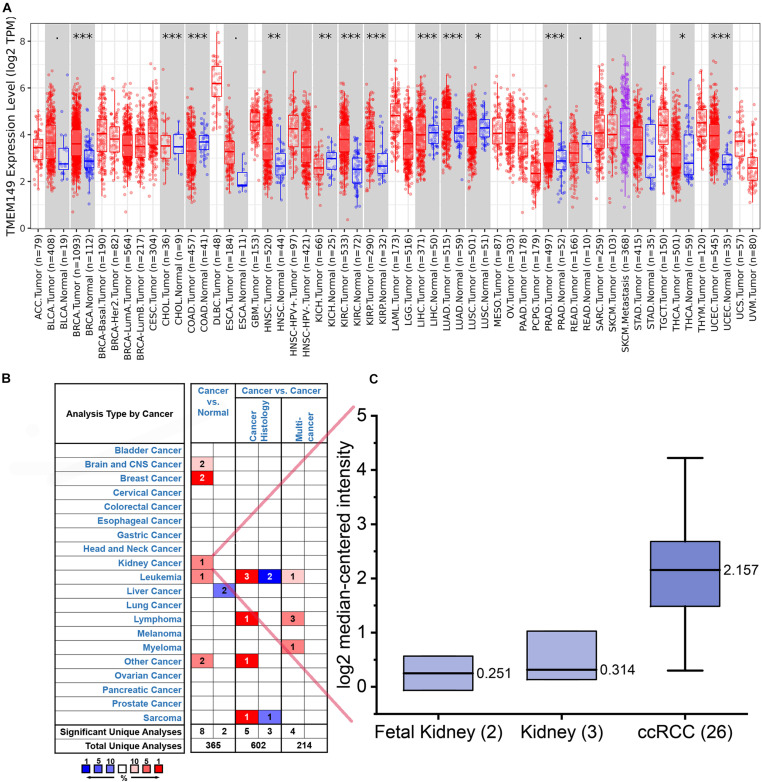
The expression level of IGFLR1 in ccRCC and pan-cancer tissue. **(A)** The differential expression of IGFLR1 in different cancer and normal tissues using TIMER online platform. The box plot showed the statistical significance of differential expression assessed by Wilcoxon test (n.s.: not significant, *p < 0.05, **p < 0.01, ***p < 0.001). **(B)** The Oncomine database showed the high or low expression of IGFLR1 in various cancer tissues compared to normal tissues. The threshold in Oncomine is determined according to the following value: *p* = 0.0001, fold change = 2, rank = top 10%. (NOTE: An analysis may be counted in more than one cancer type). **(C)** Comparison of IGFLR1 expression levels in normal and tumor tissues of ccRCC.

The analysis results of TIMER indicated that compared with normal tissues, IGFLR1 was up-regulated in breast invasive carcinoma, head and neck squamous cell carcinoma, ccRCC, kidney renal papillary cell carcinoma, lung adenocarcinoma, prostate adenocarcinoma, thyroid carcinoma (THCA), uterine corpus endometrial carcinoma, while down-regulated in cholangial carcinoma, colon adenocarcinoma, kidney chromophobe, liver hepatocellular carcinoma (LIHC), and lung squamous cell carcinoma (Figure 1A). As was illustrated in Figure 1B, IGFLR1 was up-regulated in brain and central nervous system cancer, breast cancer, kidney cancer, and leukaemia compared to normal tissues. Further, the box plot showed that the expression level of IGFLR1 in ccRCC was higher than that in fetal kidney and kidney significantly (2.157 vs. 0.251, 0.314, *p* < 0.05) based on literature (Yusenko et al., 2009; Figure 1C). HPA analysis showed that IGFLR1 protein was localized to the focal adhesion sites and enhanced specially in blood, liver, lymphoid tissues and HMC-1, Karpas-707, U-266/70 cell lines. What was a pity that antibody staining mainly was not consistent with RNA expression data and external verification was pending.

### Methylation Profile of IGFLR1

**FIGURE 2:**
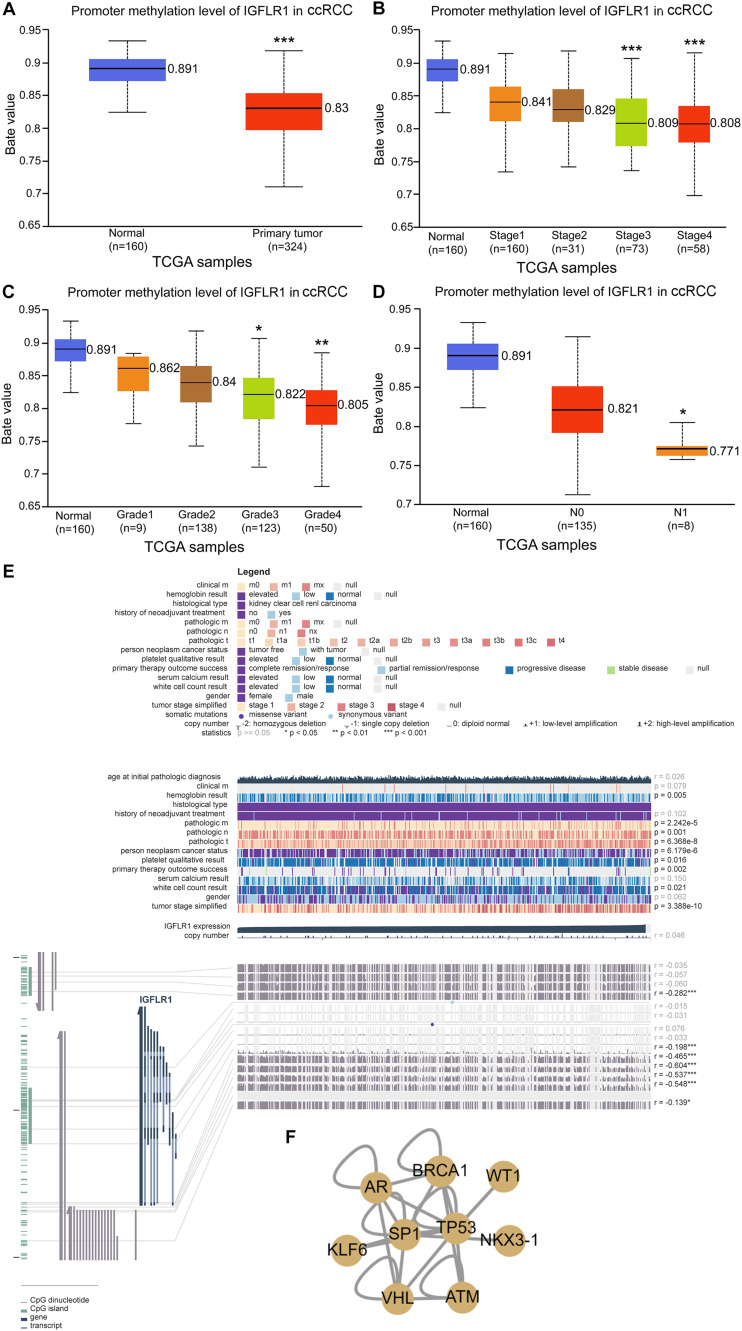
Methylation profile of IGFLR1. **(A–D)** IGFLR1 promoter methylation level in ccRCC of different sample type, stage, Grade and nodal metastasis status. All the advanced tumors were compared with stage1, Grade1 and N0 tumors. [Note: The Beta value indicates level of DNA methylation ranging from 0 (unmethylated) to 1 (fully methylated). Different beta value cut-off has been considered to indicate hyper-methylation (Beta value: 0.7–0.5) or hypo-methylation (Beta-value: 0.3–0.25)]. **(E)** The correlation between IGFLR1 expression level and DNA methylation, copy number and clinical data in ccRCC in detail. **(F)** Transcription factor (TF) regulatory network. (n.s.: not significant, * *p* < 0.05, ** *p* < 0.01, *** *p* < 0.001).

Figure 2E showed the correlation between IGFLR1 expression level and DNA methylation, copy number and clinical data in ccRCC in detail. As analyzed in the figure, IGFLR1 expression was significantly correlated with DNA methylation of CPG islands (*p* < 0.01) but not copy number (*r* = 0.046, *p*>0.05). IGFLR1 promoter methylation level was not only significantly lower in primary tumor than in normal tissues of kidney (0.891 vs. 0.83, *p* < 0.001) (Figure 2A), but also revealed a strong correlation with clinical prognosis of ccRCC. With the progression of pathological stage and histological grade and lymph node metastasis of ccRCC, the methylation level of IGFLR1 promoter decreased (Figures 2B–D). Transcription factor (TF) regulatory network based on literatures indicated that IGFLR1 could be activated by AR and repressed by NKX3-1, TP53, VHL and WT1, while the regulatory effects of other TF, including BRCA1, KLF6, SP1 and ATM, on IGFLR1 were unknown (Figure 2E).

### Prognostic Potential of IGFLR1 in ccRCC

**FIGURE 3:**
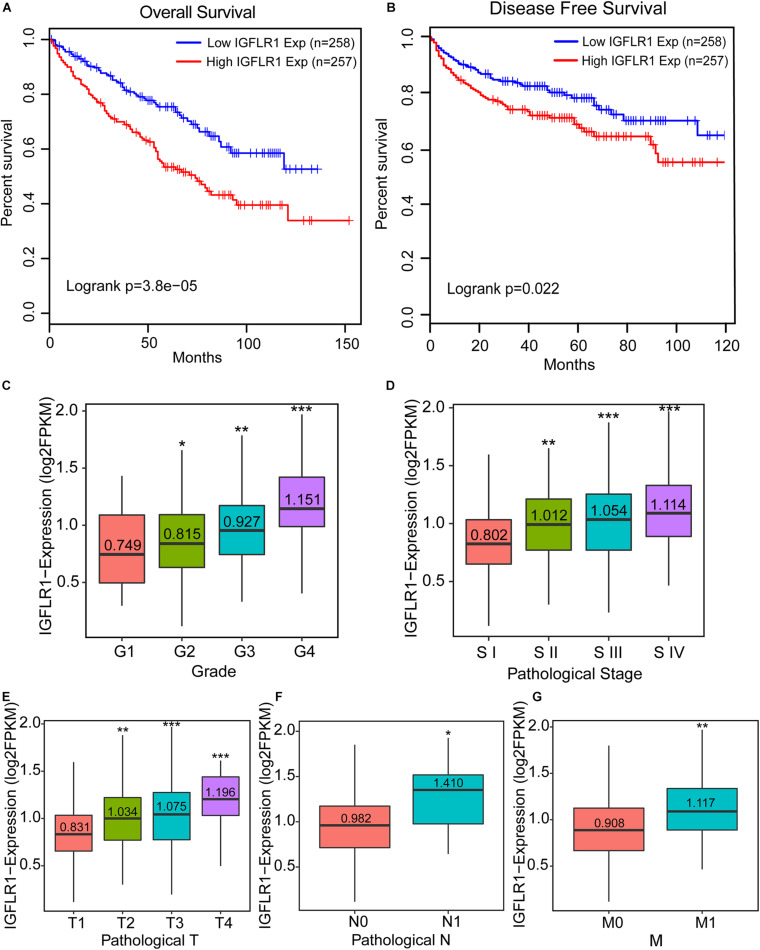
Prognostic potential of IGFLR1 in ccRCC. **(A,B)** Survival curves of OS and DFS in ccRCC (*n* = 515). We used log-rank test to analyze IGFLR1 expression in ccRCC with 50% as cutoff value to generate survival curves including OS and DFS. **(C–G)** Boxplots show the distribution of IGFLR1 expression level of ccRCC tumor samples stratified by pathologic stage, T, N, M and histological grade. All the advanced tumors were compared with stage1, Grade1, N0, T1, and M0 tumors. OS: overall survival; DFS: disease free survival. (Median of expression level were compared using independent group *t* tests when the data were normally distributed; otherwise, the Wilcoxon test was used (n.s.: not significant, *p < 0.05, **p < 0.01, ***p < 0.001).

The results of Kaplan-Meier survive analysis showed that the higher expression level of IGFLR1 was associated with poor prognosis in ccRCC (OS: Log-rank *p* < 0.001; DFS: Log-rank *p* = 0.022) (Figures 3A,B). The Supplementary Figure S1 showed the survival curves of IGFLR1 in other cancers. Furthermore, IGFLR1 expression level was higher in advanced cancer than in early cancer (stage IV vs. stage I: 1.114 vs. 0.802, *p* < 0.001; T4 vs. T1: 1.196 vs. 0.831, *p* < 0.001; N1 vs. N0: 1.410 vs. 0.982, *p* = 0.012; M1 vs. M0: 1.117 vs. 0.908, *p* < 0.001) as while as the same was true of histological grade (G4 vs. G1: 1.151 vs. 0.749, *p* < 0.001) (Figures 3C–G), which further confirmed the potential prognostic value of IGFLR1 for ccRCC. The analysis results of TIMER indicated that compared with normal tissues, IGFL1, the ligand of IGFLR1, was up-regulated in colon adenocarcinoma, lung adenocarcinoma, lung squamous cell carcinoma, rectum adenocarcinoma, THCA and uterine corpus endometrial carcinoma, while down-regulated in ccRCC and kidney renal papillary cell carcinoma (*p* < 0.05) (Supplementary Figure S2A). The expression level of IGFL1 was higher in ccRCC samples with IGFLR1-high-expression than that with IGFLR1-low-expression (0.025 vs. 0.008, *p* < 0.001) (Supplementary Figure S2B). However, with K-M analysis, we found that IGFL1 expression within the IGFLR1 high expression patients of ccRCC with poor survival had no significant difference in survival (*p* = 0.75) (Supplementary Figure S2C).

### Functional Inference of IGFLR1

**FIGURE 4:**
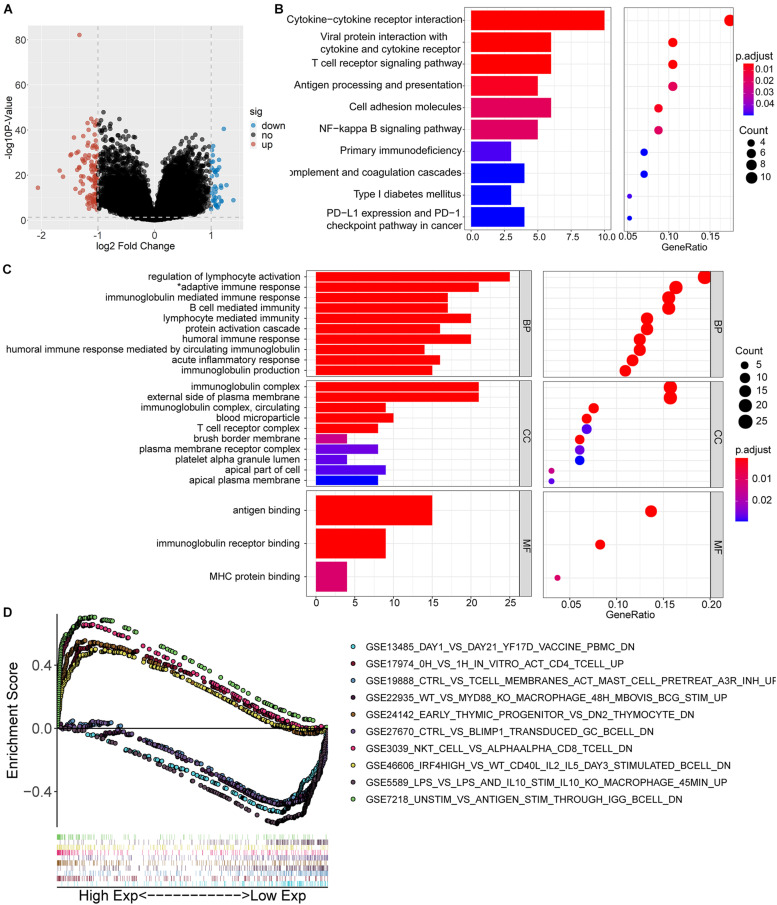
Functional inference of IGFLR1. **(A)** The volcano plot of DEGs in ccRCC patients with different IGFLR1 expression level. “down” means down-regulated DEGs; “up” means up-regulated DEGs; “no” means the difference was not statistically significant. **(B)** KEGG analysis of 152 DEGs. **(C)** GO analysis of 152 DEGs (*adaptive immune response based on somatic recombination of immune receptors built from immunoglobulin SUPERFAMILY domains). **(D)** The top 5 immunologic signature gene sets in IGFLR1 high expression group and low expression group, respectively.

Clear cell renal cell cancer tumor samples from TCGA were divided into two groups according to the expression level of IGFLR1. According to the selection criteria of DEGs |log_2_FC| > 1, FDR < 0.05, 152 genes were differentially expressed, including 112 up-regulated DEGs and 40 down-regulated DEGs in the IGFLR1-high-expression group. The volcano plot displayed the up-regulated and down-regulated DEGs in Figure 4A. To explore the cellular components, biological processes and molecular functions of IGFLR1 involved in, results of “clusterProfiler” R package revealed that the 152 DEGs were mainly related with regulation of lymphocyte activation (count = 25, *p* < 0.001), adaptive immune response based on somatic recombination of immune receptors built from immunoglobulin SUPERFAMILY domains (count = 21, *p* < 0.001), immunoglobulin mediated immune response (count = 17, *p* < 0.001), B cell mediated immunity (count = 17, *p* < 0.001), lymphocyte mediated immunity (count = 20, *p* < 0.001) and other biological processes. As well as IGFLR1 was mainly involved in immunoglobulin complex (count = 21, *p* < 0.001), external side of plasma membrane (count = 21, p < 0.001), immunoglobulin complex, circulating (count = 9, *p* < 0.001) and T cell receptor complex (count = 8, *p* < 0.001) and other cellular components. In terms of molecular functions, IGFLR1 were mainly related with antigen binding (count = 15, *p* < 0.001), immunoglobulin receptor binding (count = 9, *p* < 0.001) and MHC protein binding (count = 4, *p* < 0.001) (Figure 4C). Besides, 152 DEGs were mainly involved in Cytokine-cytokine receptor interaction (count = 10, *p* < 0.01), T cell receptor signaling pathway (count = 6, *p* < 0.01) and multiple signaling pathways (Figure 4B). Notably, GO and KEGG analysis found that IGFLR1 was significantly correlated with immune cells and molecules, and we further explored the association between IGFLR1 and immune gene set through GSEA (Figure 4D).

### Correlation Analysis Between IGFLR1 Expression and TIICs and Its Immune Marker Sets

**FIGURE 5:**
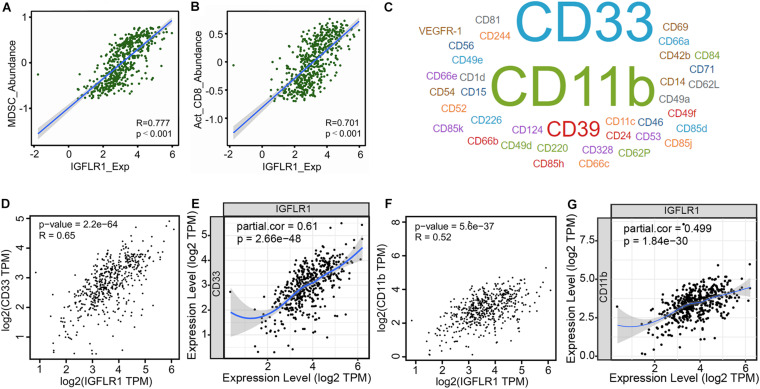
Correlation analysis between IGFLR1 expression and TIICs and its immune marker sets. **(A,B)** The correlation between IGFLR1 expression and the abundance of MDSC and activated CD8+ T cell in ccRCC based on TISIDB. **(C)** The marker genes of MDSC according to CellMarker, and the font size represented the amount of evidence supporting. **(D,F)** Correlation between IGFLR1 expression and CD33 and CD11b using TISIDB. **(E,G)** Correlation between IGFLR1 expression and CD33 and CD11b using TIMER. The partial correlation was conditioned on tumor purity.

The scatter diagrams displayed the correlation between IGFLR1 expression and multiple immune cells (Supplementary Figures S3, S4), in particular, the correlations with MDSC (R = 0.777, *p* < 0.001) and activated CD8+ T cell (*R* = 0.701, *p* < 0.001) were stronger (Figures 5A,B). CellMarker provided 38 MDSC marker genes, among which CD33 and CD11b were more supported (Figure 5C). Furthermore, the results of TISIDB showed that IGFLR1 was associated with marker genes of MDSC (CD33: *R* = 0.65, *p* < 0.001; CD11b: *R* = 0.52, *p* < 0.001) (Figures 5D,F). Similarly, after adjusting for correlation by tumor purity in TIMER, IGFLR1 expression level was significantly correlated with CD33 (partial cor = 0.61, *p* < 0.001) and CD11b (partial cor = 0.499, *p* < 0.001) in ccRCC (Figures 5E,G).

### Establishment and Evaluation of Prognostic Risk Models

**FIGURE 6:**
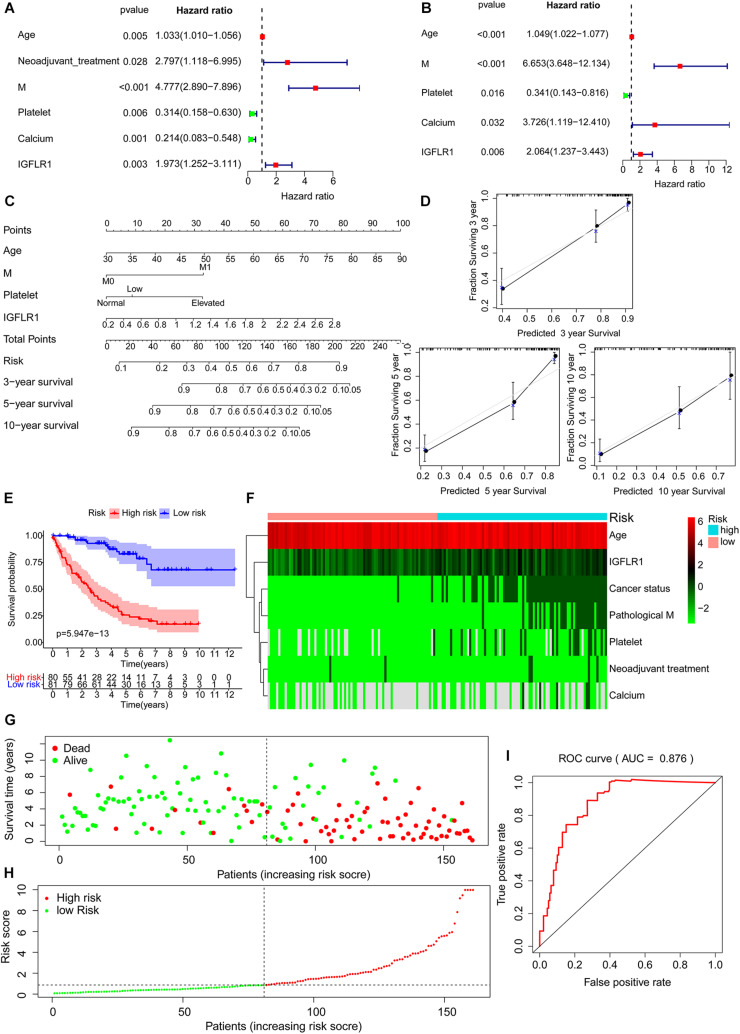
Establishment and evaluation of prognostic risk models. **(A)** Tree diagram of a univariate regression analysis. **(B)** Tree diagram of a multivariate regression analysis. **(C)** The risk score and 3, 5, 10-year survival were used by summing the points identified on the top scale for each independent covariate. **(D)** Calibration plot of actual (observed) and predicted probabilities for the Nomogram. **(E)** Survival curve based on risk score according to multivariate regression analysis. **(F)** The heatmap of prognostic factors after risk score grouping. **(G)** The plot of survival status. **(H)** The distribution of risk score. **(I)** The ROC based on risk score according to multivariate regression analysis. (The risk score was divided into high-risk group and low-risk group with a cut-off value of 50% that was 0.871). (Note: AUC range is 0∼ 1, 1 means complete consistency, 0 means complete inconsistency. It is generally believed that the prediction ability of the model is better when the AUC is greater than 0.7).

Univariate regression analysis showed age, neoadjuvant treatment, M, level of platelet, calcium and expression level of IGFLR1 were significantly correlated with the prognosis of ccRCC patients (Figure 6A). Further, multivariate regression analysis showed that the expression level of IGFLR1 was an independent prognostic factor for ccRCC (Figure 6B). Five prognostic factors including age, M, level of platelet and calcium and expression level of IGFLR1 were screened finally (*p* < 0.05), based on which a prognostic model was established and risk scores and 3, 5, 10-year survival were given for all ccRCC samples (Figure 6C). Figure 6E showed the survival curve based on the risk score, in which the survival time of patients in the high-risk group was significantly shorter than that in the low-risk group (*p* < 0.001). As shown in Figure 6F, patients in the high-risk group were older, had a history of tumor more and higher level of IGFLR1 (*p* < 0.05). Figure 6G,H showed the survival status and risk score of ccRCC patients, and more patients died in the high-risk group by the time of follow-up compared with the low-risk group. The calibration curves of 3, 5, 10-year survival and ROC of risk reflected the good predictive ability of the model (AUC = 0.876, *p* < 0.05) (Figure 6D,I).

### Analysis of Drug Sensitivity Associated With IGFLR1

**FIGURE 7:**
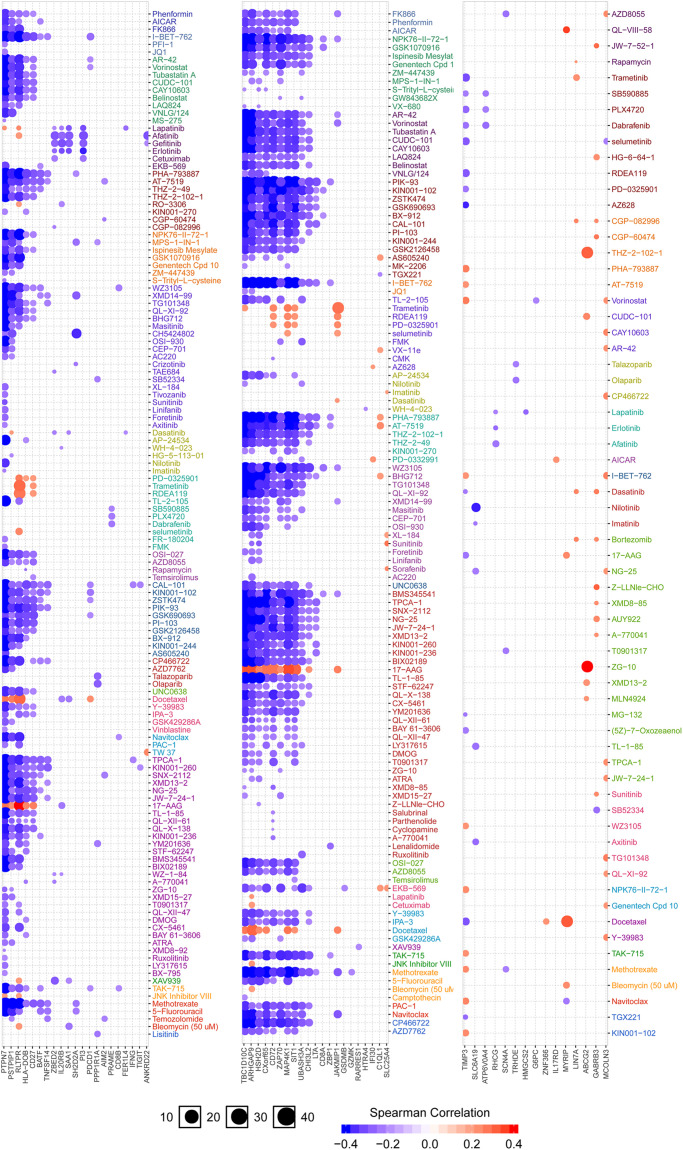
Analysis of drug sensitivity associated with IGFLR1. Red is positive correlation, which means the higher the gene expression, the more sensitive to the drug, while the blue is the opposite.

Figure 7 showed the correlation analysis between DEGs and drug sensitivity. As there were 152 DEGs, we uploaded them into 3 groups (the first two columns were up-regulated DEGs, and the last column was down-regulated DEGs) for analysis as well as the expression of each gene was performed by Spearman correlation analysis with the small molecule/drug sensitivity (IC50). The results showed that IGFLR1 was significantly associated with resistance to a variety of chemotherapeutic drugs, including methotrexate and 5-fluorouracil.

## Discussion

The present study proved that IGFLR1 was highly expressed in ccRCC, which was consistent with the previous findings (Braczkowski et al., 2016). We also attempted to further analyze the correlation between methylation and copy number variation on IGFLR1 expression level, and found that DNA methylation level may be one of the factors influencing expression, while copy number has no significant correlation. Moreover, IGFLR1 promoter methylation levels were lower in advanced ccRCC. The reason for the difference in IGFLR1 expression level in tumor samples deserved further in-depth and extensive research. Although previous studies have shown that upregulation of IGFLR1 could indicated poor OS and DFS survival in ccRCC patients (Parker et al., 2002; Schips et al., 2004; Lkhagvadorj et al., 2014), the study was limited to a small sample. And then, Mehrdad et al. evaluated IGFLR1 expression in a historical cohort of 82 ccRCC patients using the Allred score system. Survival analysis and Cox regression analysis indicated that expression of IGFLR1 was a prognostic factor in ccRCC patients, rather than an independent prognostic factor (Sichani et al., 2010). Besides, although a number of previous studies have constructed prognostic risk models based on clinical factors, gene signature, or taking both into account (Chang et al., 2018; Chen et al., 2019, 2020; Xu D. et al., 2020; Xu W.H. et al., 2020), IGFLR1 has not been reported as a factor in model construction. In the present study, we carefully examined the prognostic value of IGFLR1 in ccRCC patients using TCGA-KIRC cohort, and confirmed that the expression of IGFLR1 was an independent prognostic factor for ccRCC. Moreover, IGFLR1 expression can participate in the construction of prognostic risk model for ccRCC with age, M and level of platelet and calcium, and the models have satisfactory predictive power. Models built by different researchers eventually incorporate different factors, which may be caused by the differences in population, screening methods and data processing.

Unlike most previous studies, we also attempted to characterize the associated elevated IGFL1R and immune response, rather than limiting cases to carbohydrate metabolism (Solarek et al., 2015). Recently, a merging evidence strongly suggest that IGFLR1 may be related to the immune system, for example, Zhang et al. (2018) found that IGFLR1 was highly expressed on the surface of CD8+ T cell in CRC, which had the function of exhausting T cells. Besides, it has been previously reported that IGFLR1 expression was closely related to immune infiltration of CRC, LIHC and non-small cell lung cancer (Ren and Zhang, 2019). To further explore how IGFLR1 affects ccRCC process, we screened 152 IGFLR1-related genes through variation analysis. Pathway and gene sets enrichment analysis results showed that DEGs were enriched in the activation and regulation of immune cells and intercellular adhesion pathways. The subsequent immunocorrelates analysis indicated that IGFLR1 was strongly correlated with T cell activation and MDSC accumulation in ccRCC. Therefore, our study provided the reference for elucidating the potential role of IGFLR1 in tumor immunology and its potential application as a biomarker for ccRCC. There were sufficient evidences that the accumulation of MDSC was closely related to the poor clinical prognosis of pan-cancer (Diaz-Montero et al., 2009; Sun et al., 2012; Arihara et al., 2013; Huang et al., 2013; Zhang et al., 2013; Solito et al., 2014; Messmer et al., 2015; Angell et al., 2016; Yang et al., 2017). MDSC has been shown to produce vascular endothelial growth factor (VEGF) and basic fibroblast growth factor (bFGF) to promote tumor angiogenesis (Yang et al., 2004; Du et al., 2008; Kujawski et al., 2008). In addition to angiogenesis, MDSC also promoted tumor development through immunosuppression, targeting T cells primarily (Gabrilovich, 2017; Veglia et al., 2018). Correlation analysis revealed a strong correlation between IGFLR1 and the major factors (TGFβ, IL-10) which were associated with immunosuppression function of MDSC. This result suggested that it may be one of the potential mechanisms by which IGFLR1 promotes ccRCC tumorigenesis to promote the immunosuppressive activity of MDSC. However, studies on the relationship between IGFLR1 expression and TIICs have been limited to pedigree tracking and single-cell RNA sequencing results, while the specific mechanism has not been studied in detail.

We attempted to apply the results of this study to clinical practice, so we not only established a prognostic risk model, but also explored the correlation between IGFLR1 and tumor resistance. It’s worth noting that MDSC was eliminated with relatively low doses of gemcitabine and 5-fluorouracil (5-FU) (Suzuki et al., 2005; Le et al., 2009; Vincent et al., 2010). The results of the present analysis showed that IGFLR1 was significantly correlated with resistance of multiple chemotherapy drugs, including 5-FU, which could help clinicians select effective drugs for individual cancer patient.

Our study mainly focused on the prediction of the diagnostic, therapeutic and prognostic value of IGFLR1 in RCC from the perspective of bioinformatics. In addition, we analyzed the correlation between IGFLR1 and infiltration of TIICs and the immune-related signaling pathways. About the prediction of the diagnostic value of IGFLR1 in RCC in this study, the differentially expressed analysis results showed that IGFLR1 was significantly high expressed in RCC compared with para-cancer, which indicated that people with high expression of IGFLR1 had a higher probability of RCC. Signaling pathway analysis provided a hypothesis for exploring how IGFLR1 regulates the progress of RCC. Regarding the therapeutic value of IGFLR1, firstly, IGFLR1 can be used as a potential target, and inhibiting the expression of IGFLR1 may slow the progression of RCC tumors. In addition, methylation may be a potentially effective method to inhibit IGFLR1 expression, which provides an idea for *in vitro* experiments. Besides, IGFLR1 expression was significantly positively correlated with CD8+T cell and MDSC infiltration, suggesting that inhibition of CD8+T cell and MDSC activation may also slow down the progression of RCC. As for the prognostic value of IGFLR1, firstly, box plot showed that in RCC patients, the later tumor stage, the higher IGFLR1 expression level was. In addition, K-M survival curve showed that, patients with high IGFLR1 expression had significantly shorter survival time and worse prognosis. Besides, multivariate Cox regression analysis showed that IGFLR1 expression level was an independent prognostic factor for RCC. However, we have to say that this study is a retrospective study and has several limitations. Therefore, prospective studies with a larger sample size are called to verify the clinical application of the IGFLR1 in the personalized management of ccRCC. This study findings inferred from bioinformatics, lacking experimental verification, such as the expression of IGFLR1 in TILs was not resolved at the protein level, functional studies with isolated TIILs and blocking or interaction with the IGFLR1 was missing. As a continuation of future studies, we will supplement them in future studies.

In this study, we used RNA-Seq data and clinical data analysis of TCGA-KIRC to find that expression level of IGFLR1 was an independent prognostic factor and was significantly associated with immune signaling pathway and infiltration of MDSC in ccRCC. As well as IGFLR1 was significantly correlated with resistance of multiple chemotherapy drugs. Therefore, IGFLR1 may play an important role in tumor related immune infiltration and showed potential diagnostic, therapeutic and prognostic value in ccRCC.

## Extended Data

https://figshare.com/articles/dataset/TCGA-KIRC_RNA-seq/12980438,

https://figshare.com/articles/dataset/TCGA-KIRC_GDC_phenotype/12980435,

https://figshare.com/articles/dataset/data/12980429,

https://figshare.com/articles/journal_contribution/IGFLR1_code_R/12980417.

## Data Availability Statement

Publicly available datasets were analyzed in this study. This data can be found here: https://portal.gdc.cancer.gov/, https://www.oncomine.org/resource/login.html https://cistrome.shinyapps.io/timer/ https://portals.broadinstitute.org/ccle, http://ualcan.path.uab.edu/index.html, https://www.mxpresstrans.com/, https://www.grnpedia.org/trrust/, http://gepia.cancer-pku.cn/index.html, http://cis.hku.hk/TISIDB/index.php, http://biocc.hrbmu.edu.cn/CellMarker/, and http://bioinfo.life.hust.edu.cn/web/GSCALite/.

## Author Contributions

WS, YS, and PG were involved in conception and design of the study and revised the manuscript. XH was involved with revision of the article for important intellectual content. YY was involved with data interpretation. YZ and SH were assisted in revision and arrangement of the manuscript. LW was involved with data interpretation and revision of the article for important intellectual content. JZ was involved with revision of the article for important intellectual content, reading and approving the final version of the submitted manuscript as well as coordinating the entire process. All authors contributed to the article and approved the submitted version.

## Conflict of Interest

The authors declare that the research was conducted in the absence of any commercial or financial relationships that could be construed as a potential conflict of interest.

## Footnotes

1 https://portal.gdc.cancer.gov/

2 https://www.oncomine.org/resource/login.html/

3 https://cistrome.shinyapps.io/timer/

4 https://portals.broadinstitute.org/ccle

5 https://www.proteinatlas.org/

6 http://ualcan.path.uab.edu/index.html

7 https://www.mxpresstrans.com/

8 https://mexpress.be/about.html

9 https://www.grnpedia.org/trrust/

10 http://gepia.cancer-pku.cn/index.html

11 https://www.gsea-msigdb.org/gsea/index.jsp

12 http://cis.hku.hk/TISIDB/index.php

13 http://biocc.hrbmu.edu.cn/CellMarker/

14 http://bioinfo.life.hust.edu.cn/web/GSCALite/
