# Supplementary material for: IGFLR1 as a Novel Prognostic Biomarker in Clear Cell Renal Cell Cancer Correlating With Immune Infiltrates
Source: Front Mol Biosci. 2020 Nov 26;7:565173. doi: 10.3389/fmolb.2020.565173 (PMC7726438; doi:10.3389/fmolb.2020.565173)
Supplement: Supplementary file 1 [file Table_1.DOCX]

Supplementary Material

# Supplementary Figures
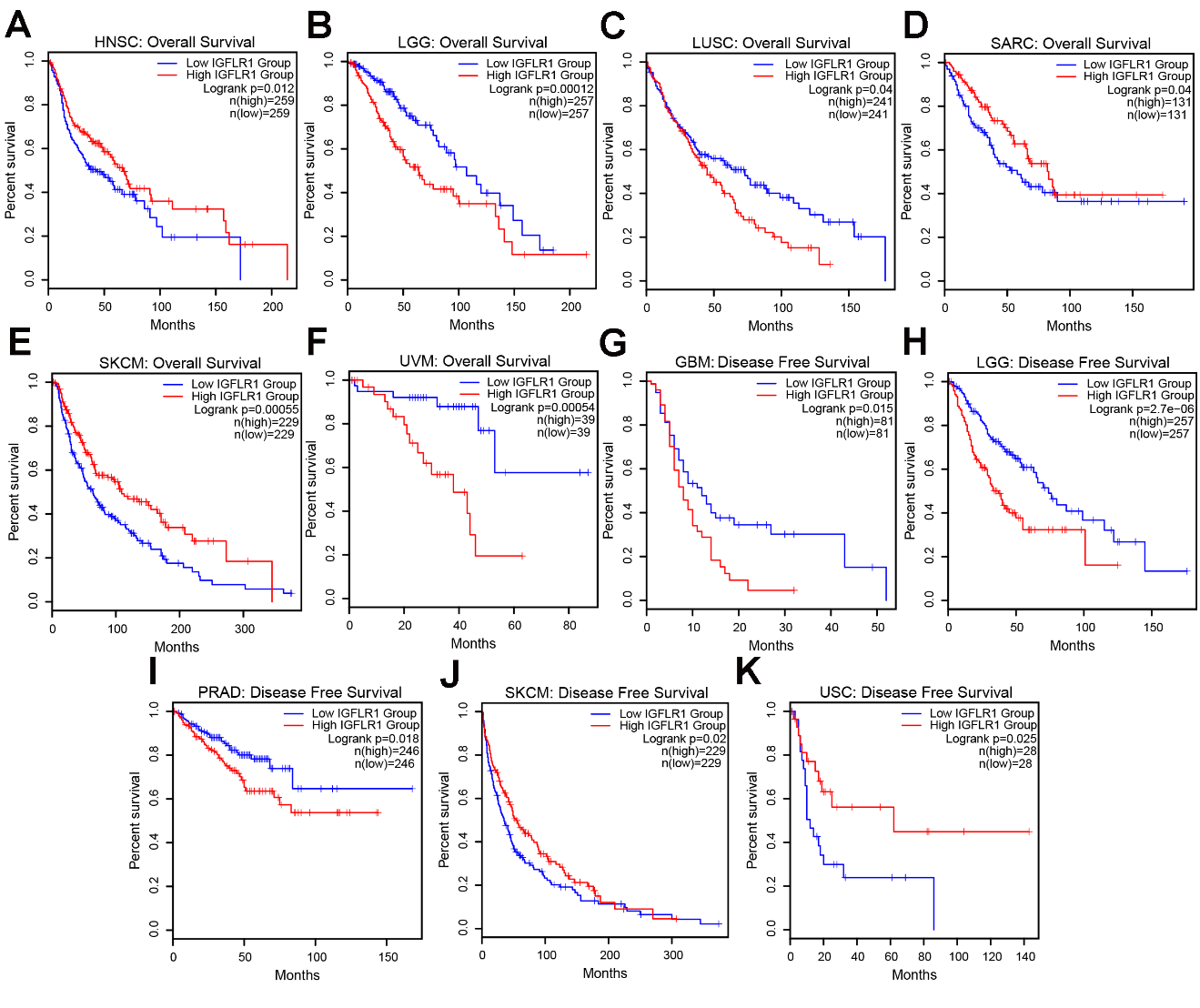


**Supplemental Figure. 1** The Kaplan-Meier survival curves showed the effect of the expression level of IGFLR1 on the survival time of patients with different types of cancers in the GEPIA. (A-F) Survival curves of OS in head and neck squamous cell carcinoma (HNSC), brain lower grade glioma (LGG), lung squamous cell carcinoma (LUSC), sarcoma (SARC), skin cutaneous melanoma (SKCM), uveal melanoma (UVM). (G-K) Survival curves of DFS in glioblastoma multiforme (GBM), brain lower grade glioma (LGG), prostate adenocarcinoma (PRAD), skin cutaneous melanoma (SKCM), uterine carcinosarcoma (USC). OS, overall survival; DFS, disease free survival. DOI: <https://figshare.com/articles/figure/Fig_S1/12980465>


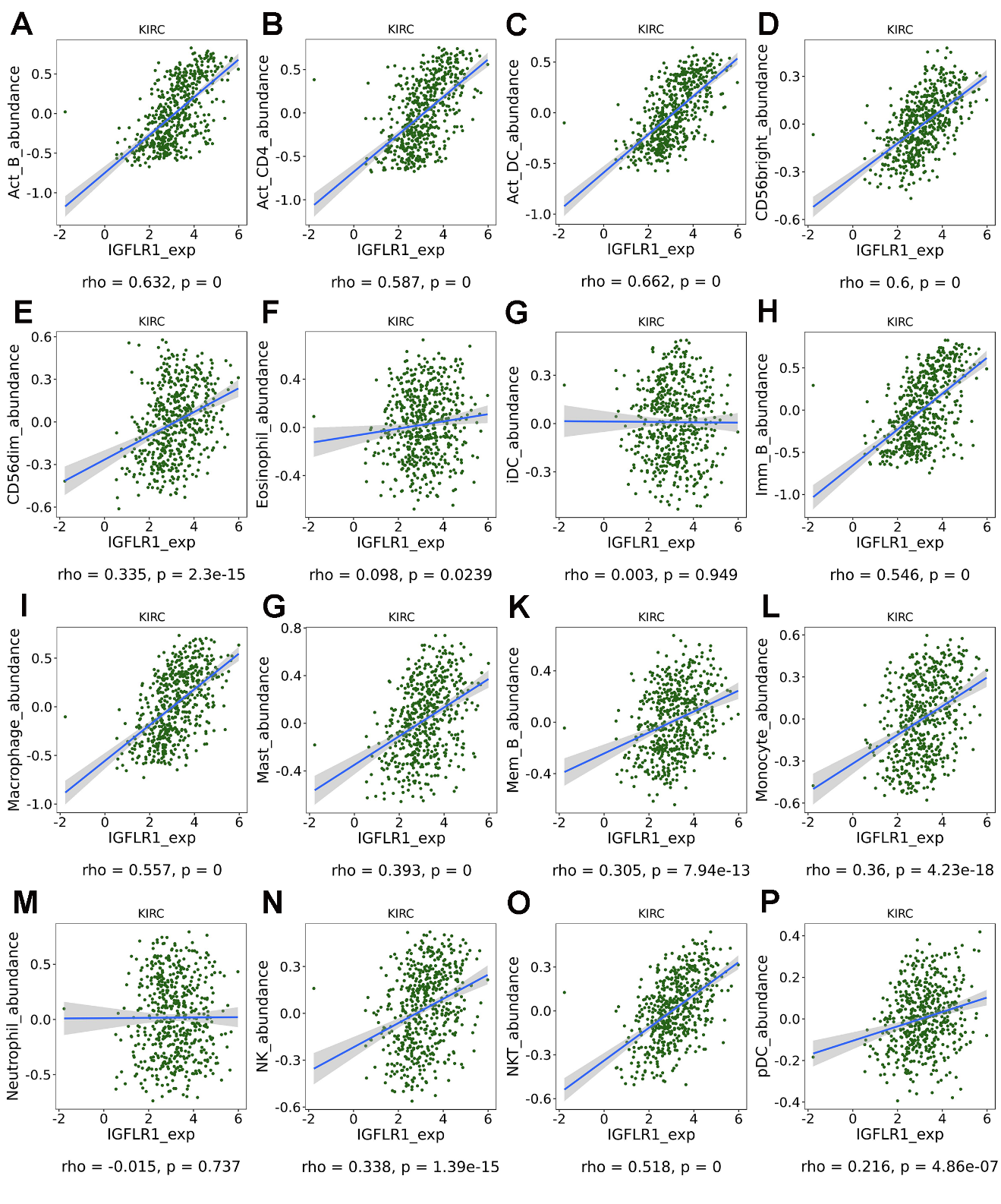


**Supplemental Figure. 2** The correlation between IGFLR1 expression and the abundance of TIICs of ccRCC in TISIDB. (A) Activated B cell, (B) Activated CD4 T cell, (C) Activated dendritic cell, (D) CD56bright natural killer cell, (E) CD56dim natural killer cell, (F) Eosinophil, (G) Immature dendritic cell, (H) Immature B cell, (I) Macrophage, (J) Mast cell, (K) Memory B cell, (L) Monocyte, (M) Neutrophil, (N) Natural killer cell, (O) Natural killer T cell, (P) Plasmacytoid dendritic cell. DOI: <https://figshare.com/articles/figure/Fig_S2/12980423>


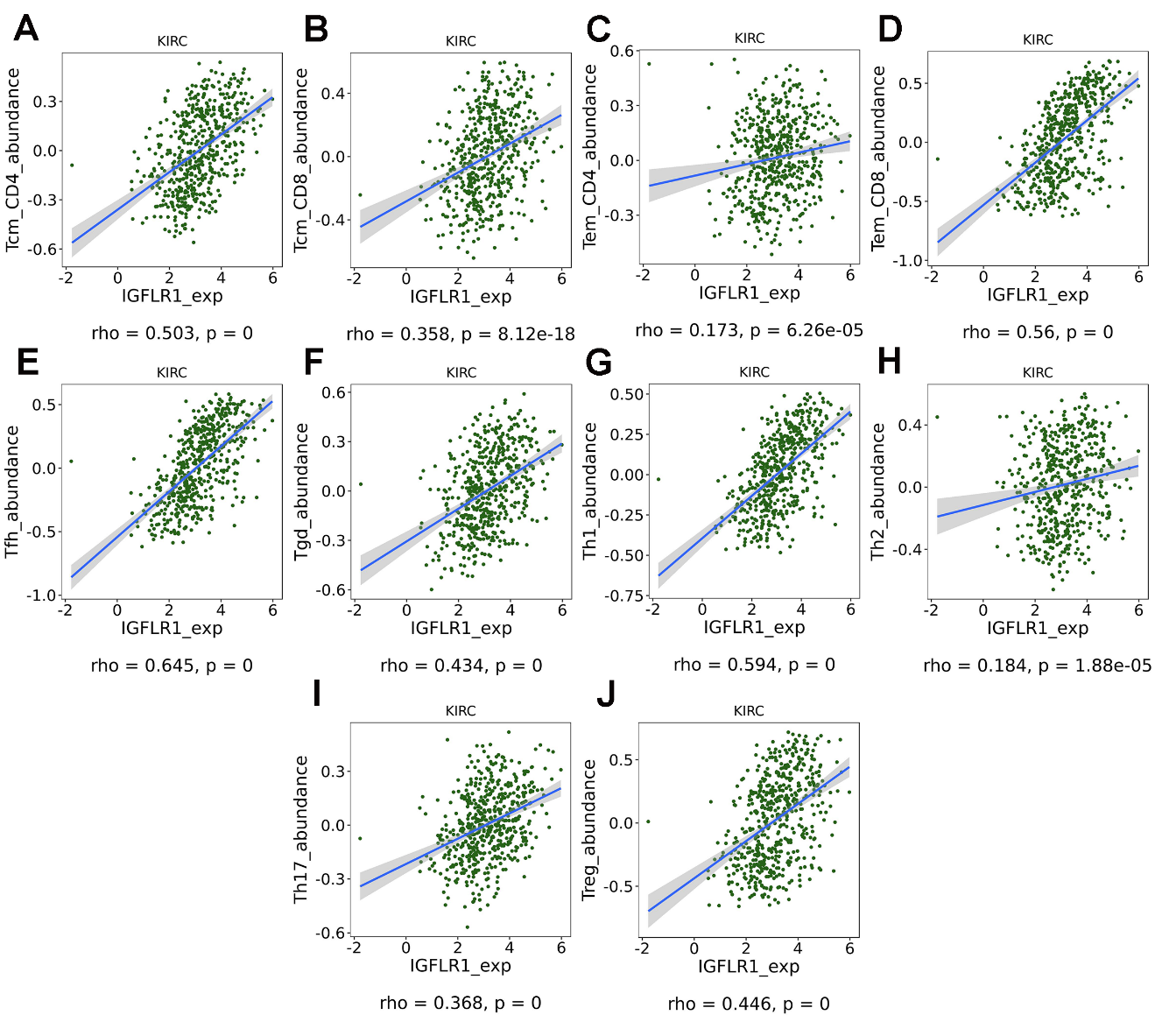


**Supplemental Figure. 3** The correlation between IGFLR1 expression and the abundance of TIICs of ccRCC in TISIDB. (A) Central memory CD4 T cell, (B) Central memory CD8 T cell, (C) Effector memory CD4 T cell, (D) Effector memory CD8 T cell, (E) T follicular helper cell, (F) Gamma delta T cell, (G) Type 1 T helper cell, (H) Type 2 T helper cell, (I) Type 17 T helper cell, (J) Regulatory T cell. DOI: <https://figshare.com/articles/figure/FigS3/12980426>


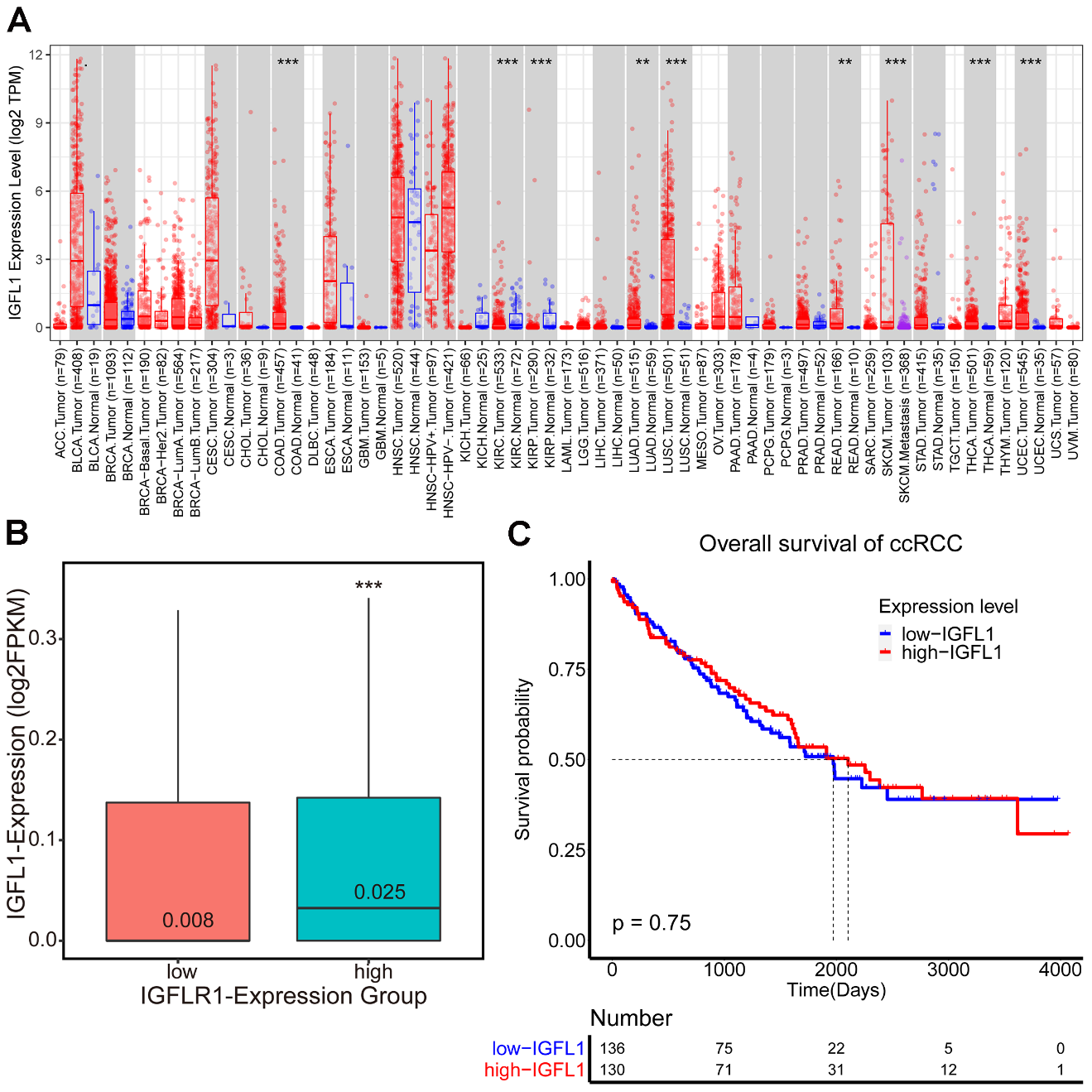


**Supplemental Figure. 4** Expression and survival analysis of IGFL1. (A) The expression of IGFL1, the ligand of IGFLR1, in general carcinoma was analyzed by using TIMER database. (B) The expression level of IGFL1 in the samples with high expression of IGFLR1 and low expression of IGFLR1. (C) The influence of the expression level of IGFL1 in the samples with high expression of IGFLR1 on the prognosis of ccRCC patients. (n.s.: not significant, * *p* < 0.05, ** *p* < 0.01, *** *p* < 0.001.) DOI: https://figshare.com/articles/figure/Fig_S4/13027823
